# Impact of sex and role of coronary artery disease in out-of-hospital cardiac arrest presenting with refractory ventricular arrhythmias

**DOI:** 10.3389/fcvm.2023.1074432

**Published:** 2023-04-11

**Authors:** Maria Luce Caputo, Enrico Baldi, Joel Daniel Krüll, Damiano Pongan, Ruggero Cresta, Claudio Benvenuti, Roberto Cianella, Roberto Primi, Alessia Currao, Sara Bendotti, Sara Compagnoni, Francesca Romana Gentile, Luciano Anselmi, Simone Savastano, Catherine Klersy, Angelo Auricchio

**Affiliations:** ^1^ Cardiology Department, Cardiocentro Ticino Institute, Lugano, Switzerland; ^2^ Division of Cardiology, Fondazione IRCCS Policlinico San Matteo, Pavia, Italy; ^3^ Fondazione Ticino Cuore, Lugano, Switzerland; ^4^ Federazione Cantonale Ticinese Servizi Autoambulanze, Bellinzona, Switzerland; ^5^ Department of Molecular Medicine, University of Pavia, Pavia, Italy; ^6^ Service of Clinical Epidemiology and Biostatistics, Fondazione IRCCS Policlinico San Matteo, Pavia, Italy

**Keywords:** OHCA, shockable rhythm, refractory ventricular arrhythmias, sex, coronary artery disease

## Abstract

**Introduction:**

There are limited data on sex-related differences in out-of hospital cardiac arrests (OHCAs) with refractory ventricular arrhythmias (VA) and, in particular, about their relationship with cardiovascular risk profile and severity of coronary artery disease (CAD).

**Purpose:**

Aim of this study was to characterize sex-related differences in clinical presentation, cardiovascular risk profile, CAD prevalence, and outcome in OHCA victims presenting with refractory VA.

**Methods:**

All OHCAs with shockable rhythm that occurred between 2015 and 2019 in the province of Pavia (Italy) and in the Canton Ticino (Switzerland) were included.

**Results:**

Out of 680 OHCAs with first shockable rhythm, 216 (33%) had a refractory VA. OHCA patients with refractory VA were younger and more often male. Males with refractory VA had more often a history of CAD (37% vs. 21%, *p* 0.03). In females, refractory VA were less frequent (M : F ratio 5 : 1) and no significant differences in cardiovascular risk factor prevalence or clinical presentation were observed. Male patients with refractory VA had a significantly lower survival at hospital admission and at 30 days as compared to males without refractory VA (45% vs. 64%, *p* < 0.001 and 24% vs. 49%, *p* < 0.001, respectively). Whereas in females, no significant survival difference was observed.

**Conclusions:**

In OHCA patients presenting with refractory VA the prognosis was significantly poorer for male patients. The refractoriness of arrhythmic events in the male population was probably due to a more complex cardiovascular profile and in particular due to a pre-existing CAD. In females, OHCA with refractory VA were less frequent and no correlation with a specific cardiovascular risk profile was observed.

## Introduction

In Europe, approximately 400.000 people suffer from an out-of-hospital cardiac arrest (OHCA) each year (1). Although a wide discrepancy in survival has been reported among different countries (2), some aspects in clinical presentation, including occurrence in a public location, younger age, witness status and shockability of the first rhythm correlate with higher odds of survival (3, 4). Among patients with initial shockable rhythm, there is a sizable group of patients who may require multiple shocks to terminate ventricular arrhythmias (VA) (5). About 20% of OHCAs with a shockable rhythm show refractory VA, defined as a fast ventricular rhythm which requires >3 consecutive shocks to be terminated (5–8). This cohort represents a very challenging OHCA subgroup because of its high mortality rate and the limited data available regarding management (9), since most patients die before reaching the hospital, and thus no diagnostic work up is possible. Coronary artery disease (CAD) is the main underlying disease in OHCA with first shockable rhythm and acute myocardial infarction (MI) accounts for 60% of all cardiac arrests (10). In male patients, in whom acute CAD is significantly more frequent, the proportion of OHCA presenting with VA is even higher (11). However, it is not known if there is always a correlation between acute CAD and refractory VA and if there are sex-related differences. Yannopoulos et al. reported a significant coronary artery stenosis in more than 80% of patients of which about 60% were cause by acute thrombosis (12). However, if this observation holds true for both male and female patients and if there are sex-specific differences in terms of cardiovascular profile and coronary disease burden is largely uninvestigated.

Aim of this study was to assess sex-related differences in clinical presentation, cardiovascular risk profile, CAD prevalence, and outcome in OHCA victims presenting with refractory VA.

## Methods

### Study design and setting

This study is a retrospective analysis of all prospectively collected data of OHCAs with shockable rhythm that occurred between 2015 and 2019 in the province of Pavia, northern Italy (Cardiac Arrest Registry of the Lombardy Region—Lombardia CARe), and in the Canton Ticino, southern Switzerland (Ticino Region Cardiac Arrest Registry—TiReCA). In both regions, several initiatives were brought forward to reduce intervention times in OHCA victims, including time to first cardio-pulmonary resuscitation (CPR) and defibrillation (13, 14). Both registries and resuscitation networks were previously described (13–16). They follow the Utstein recommendations to collect OHCA data (17, 18) and were approved by the local ethical committees. Both regions have similar demographic features as well as key aspects of the resuscitation network organization, including an optimization program for automatic and public external defibrillators (AED/PAD) throughout the territory and a well-defined protocol to guarantee prompt and fast access to urgent coronary angiography for OHCAs with shockable rhythms. Moreover, in both regions those patients requiring urgent coronary angiography are customarily centralized in one reference hospital.

### Participants

All consecutive patients with OHCA of medical origin and a shockable rhythm, in which resuscitation was attempted between 1st of January 2015 and 31st of December of 2019, were considered for inclusion in the study. Patients with missing data were excluded from further analysis.

### Definitions

*Refractory VA* was defined as ventricular fibrillation (VF) or unstable ventricular tachycardia (VT) persisting despite three shocks (5–8). *Survival to hospital admission* was defined as sustained return of spontaneous circulation (ROSC) at arrival at the emergency department. This definition corresponds to the Utstein recommendations’ core outcome “survived event” (17). 30-day survival was defined as patient alive 30 days after OHCA (17). Neurological outcome was evaluated with the cerebral performance category (CPC) score (17). CPC 1 and 2 were considered as good neurological outcome. CAD was assessed with coronary angiography. A coronary artery lesion with an obstruction of ≥50% in the left main coronary artery (LMCA), or of ≥75% in the other coronary vessels was considered significant. CAD involving LMCA plus right coronary artery (RCA) was considered equivalent to a three-vessel disease (19).

### Statistical analysis

We used Stata 17 (StataCorp, College Station, TX, USA) for all analyses. We described continuous variables with the median and 25th–75th percentiles (IQR) if continuous, and with counts and percent if categorical. We compared groups respectively with the Mann Whitney U test and the Fisher exact test. A multivariable logistic regression analysis was performed to assess independent predictors of survival at hospital admission and at 30-days. Potential predictors of outcome in the analytic model were selected based on biological plausibility and data reported in previous studies and included: sex (male/female), age (5-years groups), basic life support (BLS) before arrival of emergency medical system (EMS) (yes/no), witness status (none, bystander, EMS), OHCA location (private, public), number of shocks delivered (1, 2–3, ≥4), delay from EMS alert to defibrillation (minutes), and delay from alert to EMS arrival (minutes). We log-transformed all delay times. We reported odds ratios and 95% confidence intervals (OR, 95%CI). A 2-sided *p*-value <0.05 was considered statistically significant.

## Results

**Figure 1:**
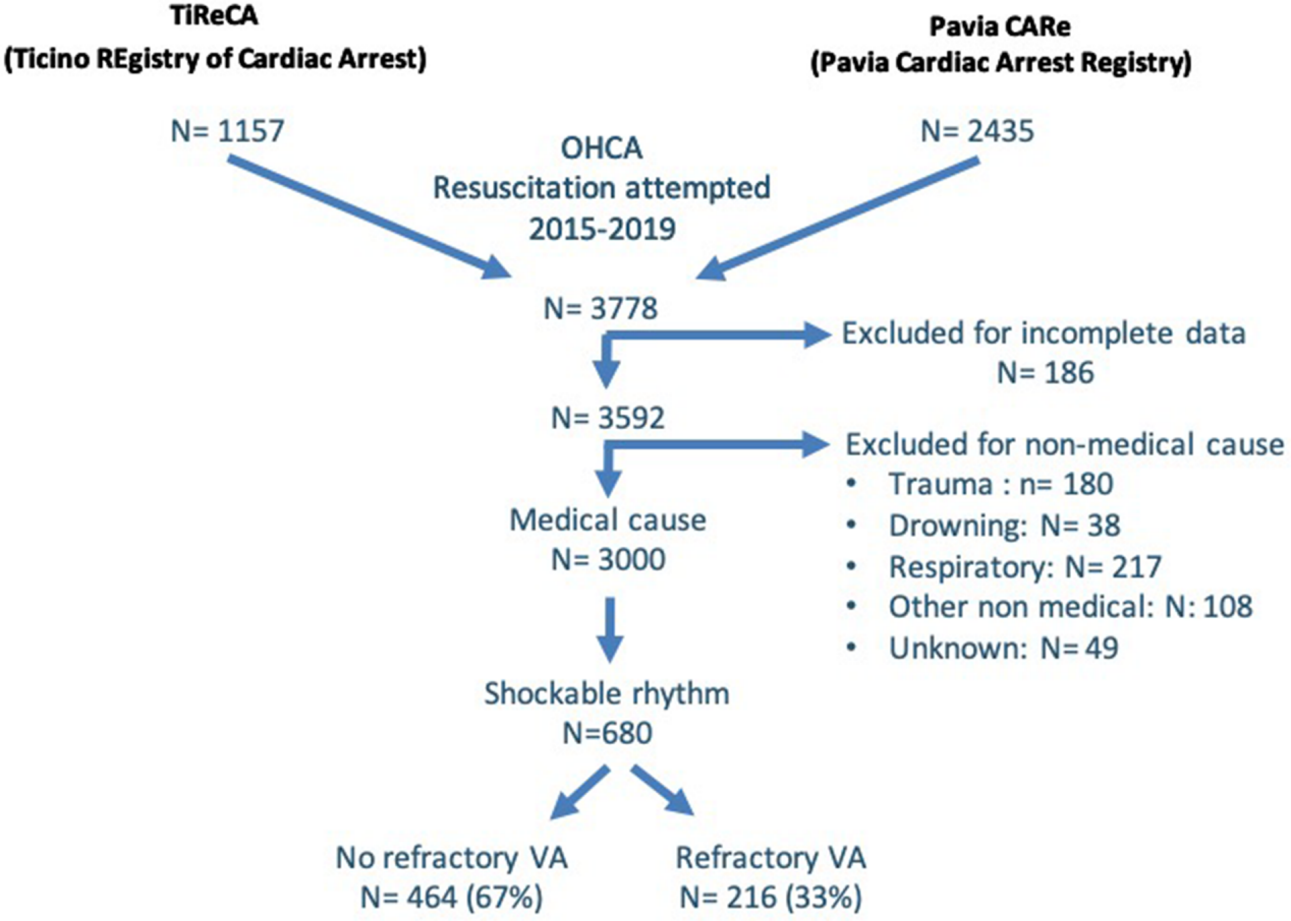
Flow chart of patients’ inclusion criteria.

The population included in the study is reported in Figure 1. During the study period, 3,778 patients had an OHCA with attempted resuscitation and were included in the two registries. A total of 186 patients had incomplete data and were excluded. Of the remaining 3,592 patients, 3,000 had an OHCA of presumed medical cause, of which 680 presented with a shockable rhythm and were included in the study.

### OHCA with refractory ventricular arrhythmias

Among patients with a first shockable rhythm, 216 (33%) had a refractory VA. Table 1 shows demographic and OHCA presentation characteristics of the two groups: patients with and without refractory VA. OHCA patients with refractory VA were younger (median age 65 [IQR 55–75] vs. 69 [IQR 59–80] years, *p* 0.003) and more often males (83% vs. 74%, *p* 0.01) as compared to patients without VA. Furthermore, their survival at hospital admission and at 30 days was significantly lower (46% vs. 64%, *p* < 0.001% and 25% vs. 48%, respectively; *p* < 0.001).

**Table 1:** Demographic and clinical characteristics of OHCA according to refractory VA presentation or not.

	No Refractory VA *N* = 464	Refractory VA *N* = 216	*p* value
Age, median (IQR)	69 (59–80)	65 (55–75)	0.003
Male sex, *n* (%)	344 (74)	179 (83)	0.01
Location, *n* (%)			0.35
Private	328 (71)	160 (74)	
Public	136 (29)	56 (26)	
Bystander witnessed, *n* (%)	301 (81)	158 (84)	0.39
CPR before ambulance arrival, *n* (%)	311 (67)	151 (70)	0.45
Time of ambulance arrival, min (IQR)	10.1 (7.5–13.5)	9.5 (7.3–13.0)	0.48
Time of defibrillation, min (IQR)	12 (8–17.6)	12.3 (9.0–15.7)	0.61
Survival at hospital admission, *n* (%)	299 (64)	100 (46)	<0.001
Survival at 30 days, *n* (%)	221 (48)	54 (25)	<0.001
Good CPC (1–2), *n* (%)	175 (91)	35 (81)	0.09

EMS: emergency medical system; CPR: cardio-pulmonary resuscitation; CPC: cerebral performance category.

### Cardiovascular comorbidities and severity of CAD in OHCA survivors admitted to urgent coronary angiography

A total of 285 patients (42% of the entire population) underwent urgent coronary angiography. Table 2 shows comorbidities**,** CAD prevalence and lesions distribution of patients with and without refractory VA. Cardiovascular risk profile was similar as well as clinical presentation. Moreover, number of coronary vessels involved and severity of CAD were similar between both groups.

**Table 2:** Comorbidities, cardiovascular risk factors and distribution and severity of CAD in OHCA survivors admitted to urgent coronary angiography.

	No refractory VA *N* = 221	Refractory VA *N* = 64	*p* value
**Comorbidities**			
Diabetes, *N* (%)	31 (14)	7 (11)	0.53
Arterial Hypertension, *N* (%)	114 (52)	36 (57)	0.45
Clinical history of Heart Failure, *N* (%)	18 (8)	7 (11)	0.48
Clinical history of CAD, *N* (%)	46 (21)	21 (33)	0.05
Previous atrial fibrillation, *N* (%)	22 (10)	10 (16)	0.21
Stroke, *N* (%)	7 (3)	4 (6)	0.27
Multiple comorbidities^*^, *N* (%)	69 (31)	25 (40)	0.22
Malignancy, *N* (%)	15 (7)	3 (5)	0.54
Acute coronary syndrome at ECG, *N* (%)	160 (72)	39 (61)	0.08
**Coronary disease severity, *N* of involved vessels (%)**			
Normal coronary angiography	28 (14)	13 (19)	0.45
1	80 (38)	20 (29)	0.42
2	51 (24)	18 (27)	0.23
3 or equivalent	50 (24)	17 (25)	0.85
**Coronary disease distribution, *n* (%)**			
LMCA	16 (9)	5 (9)	0.92
LAD	137 (73)	41 (70)	0.74
LCX	84 (45)	31 (53)	0.29
RCA	96 (51)	31 (54)	0.76

CAD, coronary artery disease;

STEMI, ST elevation myocardial infarction; NSTEMI, non ST-elevation myocardial infarction; LMCA, Left Main coronary artery; LAD, Left anterior descending artery; LCA, Left circumflex artery; RCA, Right coronary artery.

^*^ This category refers to patients who more than 1 of all above mentioned comorbidities (diabetes, arterial hypertension, clinical history of Heart failure and of CAD, previous atrial fibrillation).

### Sex-related differences in OHCA presentation, cardiovascular profile and CAD prevalence

Tables 3, 4 show differences in OHCA presentation according to sex. Refractory VA was significantly more frequent in males with a male to female ratio of 5:1. Male patients with refractory VA were significantly younger than female patients (65 [IQR 54–72] vs. 71 [IQR 60–82], *p* < 0.001; Table 3) and presented more often an OHCA that was bystander witnessed (88% vs. 66%, *p* 0.01, Table 3).

**Table 3:** Sex related differences in OHCA presenting with refractory VA.

	Males *N* = 179	Females *N* = 37	*p* value
**Age, Median (IQR)**	65 (54–72)	71 (60–82)	<0.001
**Location, *n* (%)**			0.15
Private	129 (72)	31 (84)	
Public	50 (28)	6 (16)	
**Bystander witnessed, *n* (%)**	138 (88)	20 (66)	0.01
**CPR before EMS arrival, *n* (%)**	129 (72)	22 (59)	0.16
**Time of EMS arrival, Min (IQR)**	10 (7–14)	9 (7–13)	0.48
**Time Of Defibrillation, Min (IQR)**	12 (9–16)	12 (9–17)	0.80
**Survival at Hospital admission, *n* (%)**	81 (45)	19 (51)	0.58
**Survival at 30 days, *n* (%)**	43 (24)	11 (30)	0.52
**Good CPC (1–2), *n* (%)**	27 (79)	8 (89)	0.93
	*N* = 54	*N* = 14	** **
**Comorbidities** ^*^			
Diabetes, *N* (%)	12 (6)	1 (7)	0.27
Arterial Hypertension, *N* (%)	28 (52)	8 (61)	0.52
Clinical history of HF, *N* (%)	7 (14)	1 (7)	0.98
Clinical history of CAD, *N* (%)	19 (37)	2 (15)	0.19
Previous atrial fibrillation, *N* (%)	8 (16)	2 (15)	0.98
Stroke, *N* (%)	2 (4)	2 (15)	0.18
Multiple comorbidities^a^, *N* (%)	19 (38)	6 (46)	0.75
Malignancy, *N* (%)	3 (6)	1 (7)	1.00
**Acute coronary syndrome at ECG, *N* (%)**	31 (61)	8 (61)	0.98
**Coronary disease severity, *N* of involved vessels (%)**			0.12
Normal coronary angiography	11 (20)	2 (14)	
1	16 (30)	4 (28)	
2	11 (20)	7 (50)	
3 or equivalent	16 (30)	1 (8)	

CPR, cardiopulmonary resuscitation; EMS, emergency medical system; CPC, cerebral performance category (1 and 2 were defined as good CPC); HF, heart failure; CAD, coronary artery disease.

^a^ This category refers to patients with more than 1 of all above mentioned comorbidities (diabetes, arterial hypertension, clinical history of Heart failure and of CAD, previous atrial fibrillation).

^*^ Comorbidities, acute coronary syndrome at ECG and coronary disease severity prevalence were available only in patients who survived to hospital admission.

**Table 4:** Cardiovascular profile, comorbidities and OHCA presentation according to sex.

	Males			Females		
	No refractory VA *N* = 344	Refractory VA *N* = 179	*p* value	No refractory VA *N* = 120	Refractory VA *N* = 37	*p* value
**Age, median (IQR)**	67 (57–77)	65 (54–72)	0.02	76 (63–84)	71 (60–82)	0.37
**Location, *n* (%)**			0.30			0.53
Private	233 (67)	129 (72)		95 (79)	31 (84)	
Public	111 (33)	50 (28)		25 (21)	6 (16)	
**Bystander witnessed, *n* (%)**	223 (80)	138 (88)	0.04	78 (85)	20 (66)	0.04
**CPR before EMS arrival, *n* (%)**	235 (68)	129 (72)	0.37	76 (63)	22 (59)	0.67
**Time of ambulance arrival, min (IQR)**	10 (7–13)	10 (7–14)	0.66	10 (7–13)	9 (7–13)	0.60
**Time of defibrillation, min (IQR)**	11 (7–16)	12 (9–16)	0.12	14 (9–28)	12 (9–17)	0.20
**Survival at hospital admission, *n* (%)**	222 (64)	81 (45)	<0.001	77 (64)	19 (51)	0.16
**30-day survival, *n* (%)**	170 (49)	43 (24)	<0.001	51 (42)	11 (30)	0.15
**Good CPC, *n* (%)**	133 (91)	27 (79)	0.06	42 (89)	8 (89)	0.96
	*N* = 167	*N* = 54	** **	*N *= 54	*N* = 14	** **
**Comorbidities** ^*^						
Diabetes, *n* (%)	21 (12)	12 (6)	0.67	10 (18)	1 (7)	0.30
Arterial hypertension, *n* (%)	84 (50)	28 (52)	0.50	30 (55)	8 (61)	0.69
Clinical history of HF, *n* (%)	15 (9)	7 (14)	0.32	3 (5)	1 (7)	0.24
Clinical history of CAD, *n* (%)	36 (21)	19 (37)	0.03	10 (18)	2 (15)	0.80
Previous atrial fibrillation, *n* (%)	16 (10)	8 (16)	0.22	6 (11)	2 (15)	0.67
Stroke, *n* (%)	3 (2)	2 (4)	0.39	4 (7)	2 (15)	0.39
Multiple comorbidities^a^, *n* (%)	50 (30)	19 (38)	0.30	19 (35)	6 (46)	0.46
Malignancy, *n* (%)	12 (7)	3 (6)	0.76	3 (5)	1 (7)	0.24
**Acute coronary syndrome at ECG, *n* (%)**	125 (75)	31 (61)	0.05	35 (65)	8 (61)	0.82
**Normal coronary angiography, *n* (%)**	17 (10)	11 (20)	0.09	6 (12)	2 (14)	0.66
**3-vessels (or equivalent) CAD, *n* (%)**	43 (27)	16 (30)	0.67	7 (14)	1 (8)	0.43

CPR, cardiopulmonary resuscitation; EMS, emergency medical system; CPC, cerebral performance category (1 and 2 were defined as good CPC); HF, heart failure; CAD, coronary artery disease; STEMI, ST elevation myocardial infarction; NSTEMI, non ST-elevation myocardial infarction.

^a^ This category refers to patients with more than 1 of all above mentioned comorbidities (diabetes, arterial hypertension, clinical history of Heart failure and of CAD, previous atrial fibrillation).

^*^ Comorbidities, clinical history and CAD prevalence were calculated only in patients survived to hospital admission.

Table 4 reported differences in refractory vs. not refractory VA patients within the sex category. Male patients with refractory VA were significantly younger (65 [IQR 54–72] vs. 67 [IQR 57–77] years, *p* 0.02; Table 4) and more often witnessed by bystanders (88% vs. 80%, *p* 0.04; Table 4) as compared to males without refractory VA. As for cardiovascular risk factors, males with refractory VA had more often previous history of CAD (37% vs. 21%, *p* 0.03, Table 4) as compared to males without refractory VA. Female patients with refractory VA less frequently had witnessed OHCA (66% vs. 85%, *p* 0.04, Table 4). Differently from males, females did not exhibit significant differences in cardiovascular risk factor prevalence and clinical presentation.

### Predictors of survival

**Figure 2:**
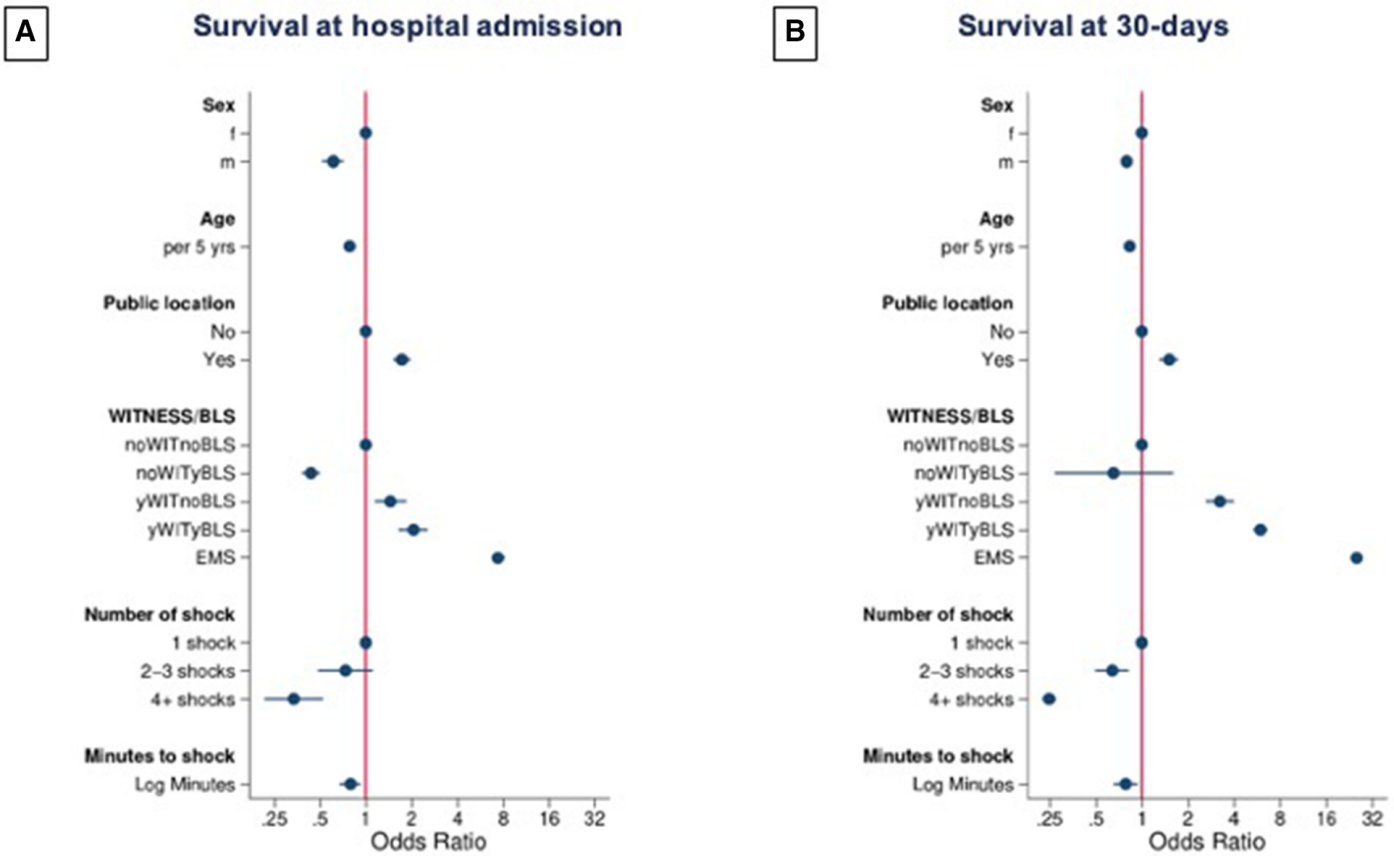
Forrest plot model of multivariable logistic regression analysis to assess independent predictors of survival. Panel A: predictors of survival at hospital admission. Panel B: predictors of survival at 30 days. Witness status and BLS before ambulance arrival were combined as follows: noWITnoBLS: bystander witnessed: no, BLS before ambulance arrival: no; noWITyBLS: bystander witnessed: no, BLS before ambulance arrival: yes; yWITnoBLS: bystander witnessed: yes, BLS before ambulance arrival: no; yWITyBLS: bystander witnessed: yes, BLS before ambulance arrival: yes; EMS: witnessed by EMS.

Female sex, OHCA in public locations, as well as bystander and EMS-witnessed OHCA, were independently associated with an increased likelihood to survive at 30 days, whereas older age, increasing number of shocks to convert the arrhythmia and delayed defibrillation affected survival negatively (Figure 2 and Table 5). In case VA was refractory, odds of survival at hospital admission and at 30 days decreased significantly (OR 0.34, 95% CI 0.21–0.53, *p* < 0.001 and OR 0.25, 95% CI 0.22–0.27, *p* < 0.001, respectively; Table 5).

**Table 5:** Multivariable logistic regression analysis to assess predictors of survival at hospital admission and at 30 days.

Survival at hospital admission			
	OR	95%CI	*p* value
**Sex**			
Female	1.0		
Male	0.61	0.52–0.72	<0.001
**Age**	0.95	0.94–0.96	<0.001
**Public Location**			
No	1.0		
Yes	1.73	1.52–1.96	<0.001
**Combined Witness/BLS before EMS arrival**			
No Wit/No BLS	1.0		
No Wit/Yes BLS	0.44	0.38–0.50	<0.001
Yes Wit/No BLS	1.46	1.15–1.85	0.002
Yes Wit/Yes BLS	2.05	1.63–2.57	<0.001
EMS Witnessed	7.44	6.71–8.25	<0.001
**Number of shocks**			
1	1.0		
2–3	0.73	0.49–1.11	0.14
4+	0.34	0.21–0.53	<0.001
**Time to defibrillation (logarithm)**	0.79	0.68–0.93	0.004
**30-day survival**			
**Sex**			
Female	1.0		
Male	0.79	0.79–0.80	<0.001
**Age**	0.96	0.96–0.97	<0.001
**Public Location**			
No	1.0		
Yes	1.50	1.29–1.74	<0.001
**Combined Witness/BLS before EMS arrival**			
No Wit/No BLS	1.0		
No Wit/Yes BLS	0.65	0.27–1.59	0.34
Yes Wit/No BLS	3.22	2.60–3.99	<0.001
Yes Wit/Yes BLS	5.95	5.33–6.64	<0.001
EMS Witnessed	25.3	23.40–27.34	<0.001
**Number of shocks**			
1	1.0		
2–3	0.64	0.49–0.82	0.001
4+	0.25	0.22–0.27	<0.001
**Time to defibrillation (logarithm)**	0.78	0.65–0.94	0.008

BLS, basic life support; EMS, emergency medical service. Witness status and BLS before ambulance arrival were combined as follows: noWITnoBLS: bystander witnessed: no, BLS before ambulance arrival: no; noWITyBLS: bystander witnessed: no, BLS before ambulance arrival: yes; yWITnoBLS: bystander witnessed: yes, BLS before ambulance arrival: no; yWITyBLS: bystander witnessed: yes, BLS before ambulance arrival: yes; EMS: witnessed by EMS.

## Discussion

The main findings of this study were: (1) OHCA presenting with refractory VA were significantly more frequent in males and in this subgroup, were associated with previous history of CAD. (2) In female patients refractory VA was rarely observed and there were no significant differences in clinical presentation, cardiovascular profile and CAD distribution between patients with and without refractory VA. (3) OHCA presenting with refractory VA had a significantly lower probability of survival despite younger age and favourable clinical presentation.

### Sex disparities in OHCA with refractory ventricular arrhythmias and role of cardiovascular risk profile and coronary artery disease

As reported by Yannopoulos et al. (20) and Choi et al. (8), we also observed a higher prevalence of refractory VA in younger and male patients (male to female ratio 5 : 1). The higher prevalence of shockable rhythms and refractory VA in male patients may be explained by the higher prevalence of CAD. It was reported that cardiac arrest may be the first clinical manifestation of an acute coronary syndrome in up to 7% of patients (21). Moreover, patients with OHCA occurring in the setting of ACS very often have a favorable presentation (witnessed status up to 85%) (22). In a large Swedish retrospective analysis of impact of sex differences on urgent coronary angiography access after OHCA with shockable rhythm, Lindgren et al. observed that male patients had more severe CAD, while females more frequently had normal coronary angiography (23). By analyzing the sex-related differences in cardiovascular risk profile and CAD in OHCA patients presenting with refractory as compared to non-refractory VA, we observed, very interestingly, that male patients with refractory VA had more often a previous history of CAD. It was previously reported that male patients more frequently have a previous history of CAD (23) and a higher prevalence of cardiovascular risk factors (24). However, up to now, it is not known if a similar cardiovascular profile is associated with refractory OHCA in male patients.

In recent cardiac magnetic resonance (CMR) imaging studies (25–27) in OHCA survivors, the presence of myocardial edema denoted an acute and transient arrhythmogenic substrate, with a favorable long-term arrhythmic outcome, whereas presence and extension of late gadolinium enhancement (LGE), i. e. scar tissue, was significantly associated with recurrent arrhythmic events (25). This is in line with our findings, where in the male population with refractory VA, history of CAD, possibly with scar, was more frequent as compared to male patients without refractory VA. Myocardial scar tissue represents a significant more challenge substrate as compared to acute myocardial ischaemia due to sudden coronary occlusion. The presence of an underlying chronic irreversible or unmodifiable cause may probably explain the refractoriness of arrhythmias in these patients and their very poor outcome.

However, while in males the refractoriness of OHCA may be explained by a more complex and unmodifiable arrhythmia substrate, the underlying mechanism in females with refractory VA remains unclear. In our population, refractory VA was quite rare in female patients and there were no significant differences as compared to female OHCA victims without refractory VA, except the more frequent unwitnessed status.

In our patient cohort, CAD represented the most frequent underlying disease in both refractory and non-refractory VA in female OHCA patients. Interestingly, compared to male patients, female patients with refractory OHCA did not show a higher mortality. A recent meta-analysis by Feng et al. (28), investigating sex-related differences in OHCA survival, confirmed a higher survival rate in women, even in postmenopausal patients. This may be due to other potential protective mechanisms independent of the effects of sex hormones. Indeed, studies on hormone-independent neuroprotective mechanisms in women with stroke found different ischemic cell death pathways in the brain (29, 30).

### Impact of refractory VA on survival at hospital admission and at 30 days

In our population, only 25% of the patients with refractory VA were alive at 30 days. This was also the case even in patients with witnessed OHCA and prompt CPR with defibrillation before EMS arrival. We observed that occurrence of refractory VA reduced the odds of survival to one-third. The very poor prognosis is even more dramatic considering that these patients were in median 5 years younger than those without refractory VA at presentation. Similarly, Holmen et al. (6) observed that 30-day survival decreased by 10% with each further shock necessary, regardless of witness status. Moreover, Hasegawa et al. (31) reported that the survival rate was 35% among witnessed OHCAs with VA as presenting rhythm treated successfully with a single shock, whereas an increased number of shocks was associated with a lower survival rate.

Altogether, these observations highlight the need for new protocols to manage out-of-hospital refractory cardiac arrests and integration of standard advanced cardiac life support (ACLS) protocols. The implementation of ECMO-facilitated resuscitation for patients with refractory ventricular fibrillation/ventricular tachycardia OHCA are encouraging but their impact on survival with a good neurological outcome compared to standard ACLS resuscitation are still controversial (32), mainly because different inclusion criteria (including definition of refractory OHCA) were applied in the studies. A deeper understanding of mechanisms of refractory VA, including sex-specific factors, might be helpful for a more tailored treatment of these patients and for a proper selection of candidates for more advanced resuscitation efforts in order to increase survival with good neurological outcome.

## Limitations

This study has some limitations. Due to the observational study design, it is not possible to exclude uncontrolled confounders, including patients' baseline medication and in-hospital treatment, which may affect survival. Moreover, the actual prevalence and severity of CAD, as well as sex related prevalence of OHCA with refractory VA and survival may be in part underestimated because complete medical history and diagnostic work up were available only for those patients admitted to hospital for urgent coronary angiography. To date, autopsy in OHCA victims is not a routine procedure in neither of our regions and we currently do not systematically collect these data.

## Conclusions

OHCA presenting with refractory VA was significantly more frequent in male patients. Despite younger age and more favourable presentation, males presenting with refractory VA had a significantly lower survival. The refractoriness of the arrhythmic events in this population was associated with a pre-existing CAD. In female patients refractory VA represent an uncommon OHCA presentation. Differently from males, females with refractory VA did not exhibit a poorer prognosis and no relationship with a previous CAD was observed.

## Data Availability

The data analyzed in this study is subject to the following licenses/restrictions: the full dataset is accessible if requested. Requests to access these datasets should be directed to Catherine Klersy, klersy@smatteo.pv.it.
